# Biatrial Remodeling in Patients with Cystic Fibrosis

**DOI:** 10.3390/jcm8081141

**Published:** 2019-07-31

**Authors:** Aleksandar Dordevic, Martin Genger, Carsten Schwarz, Cesare Cuspidi, Elvis Tahirovic, Burkert Pieske, Hans-Dirk Düngen, Marijana Tadic

**Affiliations:** 1 Department of Internal Medicine and Cardiology, Charité-University-Medicine Berlin, Campus Virchow Klinikum (CVK), 13353 Berlin, Germany; 2 Department of Pediatric Pneumology, Immunology and Intensive Care Medicine, CF Center/Charité-Universitätsmedizin Berlin, 13353 Berlin, Germany; 3 Clinical Research Unit, Istituto Auxologico Italiano IRCCS, University of Milan-Bicocca, 20036 Meda, Italy

**Keywords:** cystic fibrosis, left atrium, right atrium, function, strain

## Abstract

Background: Previous studies have focused on left and right ventricular remodeling in cystic fibrosis (CF), whereas atrial function has not been assessed in detail so far. We sought to investigate left and right atrial (LA and RA) function in patients with CF. Methods: This retrospective investigation included 82 CF patients (64 survivors and 18 non-survivors) who were referred to CF department over the period of four years, as well as 32 control subjects matched by age and gender. All participants underwent an echocardiographic examination including a strain analysis, which was performed offline and blinded for groups. Results: LA and RA volume indexes were significantly higher in CF patients than in controls and were particularly high in CF non-survivors. LA conduit and reservoir functions were significantly worse in CF survivors and non-survivors, compared with control subjects. RA phasic function was not different between controls, CF survivors and non-survivors. The parameters of lung function (forced vital capacity (FVC) and forced expiratory volume in the first second (FEV1)) and the LA and RA volume indexes were predictors of mortality in CF patients. However, in a multivariate analysis, only FVC was an independent predictor of mortality in CF patients. Conclusions: Our results suggest that both atria are enlarged, but only LA function is impaired in CF patients. LA reservoir and conduit function is particularly deteriorated in CF patients. Though statistical significance was not reached due to our limited sample size, there was a trend of deterioration of LA and RA function from controls across CF survivors to CF non-survivors. LA and RA enlargement represented predictors of mortality in CF patients.

## 1. Introduction

Cystic fibrosis (CF) is the most frequent life-threatening autosomal recessive disease in the Caucasian race, occurring in 1 out of 2500 newborns. CF is caused by almost 2000 various mutations of the CF transmembrane conductance regulator gene, located on the seventh chromosome. Previously, chronic cor pulmonale was present in 70% of infants and children dying from CF [1]. However, the natural history of CF has been dramatically changed in the last few decades, the prognosis of these patients has been significantly improved, and lifespan has been significantly prolonged.

Studies conducted in the CF population have reported significant heart remodeling and, particularly, right ventricular (RV) hypertrophy and dysfunction in the advanced stages of disease [2,3,4,5,6]. These changes are usually connected with increased pulmonary pressure that develops because of increased stiffness in pulmonary circulation, which is the result of hypertrophy and hyperplasia of the arterial media [3,7,8,9]. The introduction of new echocardiographic methods such as tissue Doppler imaging improved the diagnosis of subclinical cardiac dysfunction even in patients with mild CF [10]. Nevertheless, the adoption of strain in everyday clinical practice has enabled the detection of subtle cardiac changes in mechanics, which was not previously possible. Several studies have been published on this topic, and there has been no agreement regarding the influence of CF on left ventricular (LV) and RV mechanics [11,12,13,14,15]. These investigations have been mainly focused on the RV, but they have also demonstrated subtle LV strain changes [13,15]. However, there are still many inconsistencies that need to be resolved.

Atrial remodeling is usually underestimated and neglected in patients with various cardiovascular diseases. However, left atrial (LA) enlargement and dysfunction is related with both cardiovascular and overall mortality [16]. Right atrial (RA) function has been proven to be an important predictor of outcome in patients with pulmonary hypertension [17], which is common in the CF population. There are no data regarding atrial function in CF patients, and these data potentially could explain LV and RV diastolic dysfunction and the development of cardiac-related symptoms.

The aim of the present study was to evaluate LA and RA phasic function using the strain method in CF patients. Additionally, we sought to investigate if LA and RA function and volume represent predictors of lethal outcome in CF patients.

## 2. Methodology

This is a retrospective study that involved 82 consecutive CF patients who were referred to the CF department in the period between October 2012 and December 2016. The diagnosis of CF was confirmed via a documented positive sweat chloride test or by the identification of two genetic mutations known to cause CF. Controls were recruited from the echocardiography department among the patients who were referred to a regular check-up examination, palpitations or innocent heart murmur. Control subjects were matched with the CF group by age and gender. Patients with symptoms or signs of coronary artery disease, valve heart disease more than mild, atrial fibrillation, congenital heart disease, liver or kidney failure were excluded from this study. All study participants underwent an echocardiographic examination, and one researcher (AD) performed an offline strain analysis. This investigator was blinded to which group study participant belonged (control, CF survivors and CF non-survivors). All CF patients had spirometry before their echocardiographic examination. Patients with inadequate echocardiographic images were excluded (*n* = 15).

All CF patients had regular follow-up examinations at the CF department. Our records showed that 64 CF were alive and 18 CF patients died until June 2019, when the last check-up of our database was performed.

Anthropometric measures (height, weight) and laboratory analyses (level of fasting glucose, serum creatinine and urea) were obtained from all the participants. Body mass index (BMI) and body surface area (BSA) were calculated for each subject. The study was approved by the local ethics committee.

### 2.1. Echocardiography

Echocardiographic examinations were performed by a Vivid 7 (GE Vingmed, Horten, Norway) ultrasound machine. LV diameters, posterior wall and septum thickness were measured, and relative wall thickness was calculated according to the current recommendations [18]. The LV ejection fraction (EF) was calculated by using the modified Simpson biplane method. LV mass was calculated by using the American Society for Echocardiography formula [18] and indexed for BSA.

A pulsed-wave Doppler evaluation of transmitral LV was obtained in the apical 4-chamber view according to guidelines [19]. Tissue Doppler imaging was used to get LV myocardial velocities in the apical 4-chamber view, with a sample volume placed at the septal and lateral segments of the mitral annulus during early diastole (e’). The average of the peak early diastolic relaxation velocity (e’) of the septal and lateral mitral annulus was obtained, and the E/e’ ratio was computed.

### 2.2. Assessment of Left Atrial Volumes and Strain

LA volumes (LAVs) were measured just before the mitral valve opening. The LA volume was determined according to the biplane method in the 4-and 2-chamber views, and it was indexed for BSA (left atrial volume index—LAVI) [18].

**Figure 1:**
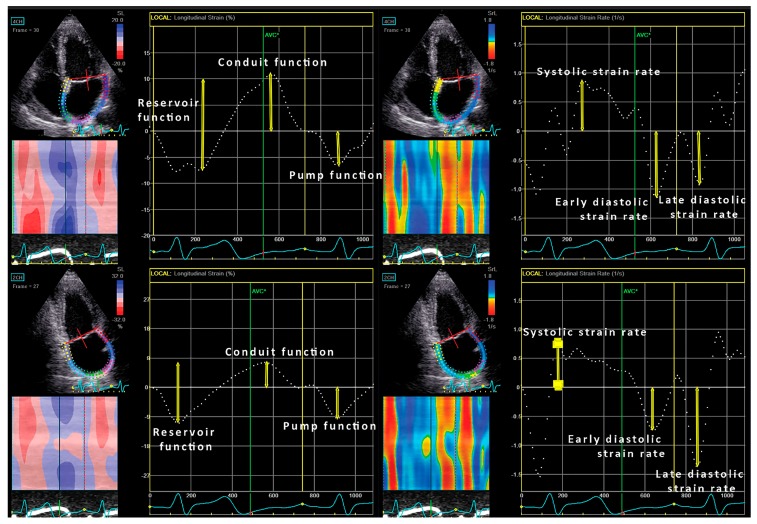
Left atrial strain and strain rate analysis in the four-chamber view (upper two plots, consequently) and in the two-chamber view (lower two plots, consequently).

2D LA strain imaging was performed in the apical 4- and 2-chamber views [20], and commercially available software Echo PAC 201 (GE-Healthcare, Horten, Norway) was used for the offline 2D strain analysis. LA strain and strain rates were calculated by P-P triggering (Figure 1). Namely, there are two methods for evaluation of LA strain: R-R and P-P triggering and we decided to use P-P triggering [20]. The LA endocardium was manually traced. LA peak strain rate was measured at the LV systolic phase, while early and late LA strain rates were measured during early LV filling and throughout the late LV diastolic phase, respectively. An average longitudinal strain curve was automatically generated, and it included a negative deflection (LA negative longitudinal strain) representing LA active contraction, followed by a positive one during LA filling (LA positive longitudinal strain). Their summation represented the total LA longitudinal strain. LA strains (positive, negative and total) were calculated by averaging the values obtained in the 4-and 2-chamber apical views.

### 2.3. Right Ventricle and Atrium

The RV internal diameter was measured in the apical four-chamber view [21]. RV global systolic function was assessed as the tricuspid annular plane systolic excursion (TAPSE) [21]. RV systolic blood pressure (PAPs) was assessed in patients with minimal/mild tricuspid regurgitation.

### 2.4. D Assessment of Right Atrial Volumes and Function

**Figure 2:**
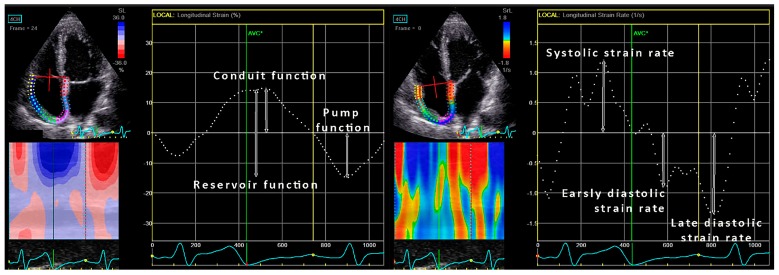
Right atrial strain and strain rate analysis in the four-chamber view.

RA volume was evaluated just before the tricuspid valve opening and indexed for BSA [4]. RA strain and strain rates were calculated by P-P triggering using the same methods that were described in the section regarding LA strain assessment (Figure 2) [21].

### 2.5. Statistical Analysis

Continuous variables are presented as mean ± standard deviation, showed a normal distribution, and were compared by the analysis of equal variance (ANOVA). An LSD post hoc analysis was used for the comparison between different groups. Differences in proportions were compared by the χ² test. Univariate and multivariate logistic regression analyses were used for the determination of predictors of mortality in the CF patients. A multivariate logistic regression analysis included variables that showed *p* < 0.1 in the univariate logistic regression analysis. The *p*-value < 0.05 was considered statistically significant.

## 3. Results

There was no significant difference in age and sex distribution between controls, CF survivors and CF non-survivors (Table 1). BMI was significantly lower in non-survivors than in controls and CF survivors. There was no difference in the prevalence in diabetes between CF survivors and CF non-survivors (Table 1). Urea and serum creatinine levels were significantly higher in CF non-survivors than in the other two groups (Table 1). The fasting glucose level was higher in CF survivors and non-survivors than in controls (Table 1). Forced vital capacity (FVC) and forced expiratory volume in the first second (FEV1) were significantly lower in CF non-survivors than CF survivors (Table 1).

### 3.1. Left and Right Ventricle

Left ventricular diameter was significantly higher in the control group than in the two CF groups (Table 2). Interventricular and posterior wall thickness did not differ between the three observed groups (Table 2). Therefore, relative wall thickness was significantly higher in the CF groups than in controls (Table 2). There was no difference in the LV mass index and the ejection fraction between the three groups. The mitral E/A ratio was lower in CF patients than in controls. Mitral E/e’ was higher in non-survivors than in controls and survivors (Table 2). Mitral deceleration time was similar between the groups (Table 2). The LA volume index was significantly higher in CF non-survivors than in controls and CF survivors (Table 2). LA dilatation (LAVI > 34 mL/m^2^) was significantly higher in CF non-survivors than in controls and CF survivors (Table 2). LV diastolic dysfunction prevalence was higher in CF non-survivors than in controls and CF survivors (Table 2).

RV diameter and areas were similar between controls and CF patients (Table 2). The parameters of RV systolic function-FAC and systolic flow velocity across the lateral segment of tricuspid annulus (s’) did not differ between CF patients and controls, whereas TAPSE was significantly lower in CF survivors and non-survivors (Table 2). The RA volume index was higher in CF patients than in controls (Table 2). There was no difference in the prevalence of RA dilatation (right atrial volume index (RAVI) > 25 mL/m^2^ for women and > 26 mL/m^2^) between controls and CF patients (Table 2). PAPs was also higher in survivors and non-survivors than in controls. Pulmonary hypertension was significantly more prevalent in CF non-survivors than in CF survivors and controls (Table 2).

### 3.2. LA and RA Strain Parameters

There was no difference in LA speckle tracking parameters between groups when global values of LA strain (average of four chamber (4Ch) and two chamber (2Ch)) were evaluated (Table 3). Different results were obtained when the LA strain was separately assessed in the 4Ch and 2Ch views. The LA positive strain measured in 4Ch, which corresponds with LA conduit function, was significantly lower in CF survivors and non-survivors (Table 3). There was no difference in the LA negative strain, which corresponds with LA active pump function (Table 3). The total LA strain and early diastolic strain rate that represent LA reservoir function were also significantly lower in CF survivors and non-survivors than in controls (Table 3). Late diastolic strain and systolic strain rates were not different among the three groups (Table 3). There was no significant difference in LA speckle tracking parameters when they were analyzed—this difference was only present in 2 Ch (Table 3).

The RA strains (total, positive and negative) and strain rates were similar between controls, CF survivors and CF non-survivors (Table 4).

### 3.3. Predictors of Mortality

Parameters of lung function (FVC and FEV1), the LA and RA volume indexes were predictors of mortality in CF patients (Table 5). PAPs was not a predictor of mortality in CF patients. However, in the multivariate analysis that included BMI, FVC, LAVI and RAVI, only FVC was an independent predictor of mortality in CF patients (OR 0.94, 95%CI: 0.90–0.98, *p* = 0.004).

## 4. Discussion

Our investigation provided several important findings: (i) Both atria were significantly enlarged in CF patients—particularly in CF non-survivors; (ii) LA conduit and reservoir functions were significantly lower in CF survivors and non-survivors than in controls; (iii) RA function was not impaired in CF patients; (iv) the atrial volume indexes (LAVI and RAVI) were predictors of mortality in CF patients.

Most investigations about CF performed in the last three decades have been focused on RV structure, RV function, and, more recently, RV mechanics [10,12,14]. LV remodeling became an interesting topic only when an echocardiographic technique developed enough to provide information other than the LV diameters and the ejection fraction. Nevertheless, LA and RA size and function have not been considered as important in CF patients. Considering the fact that bi-atrial enlargement and function are predictors of outcome in both the general population and in patients with pulmonary hypertension [16,17], it would be reasonable to hypothesize that LA and RA remodeling is also present in CF patients. A similar study has not been previously performed, and there are no data regarding atrial function and its influence on survival in CF patients.

Our findings revealed significant LA and RA enlargement in CF patients. Namely, LA and RA volumes gradually increased from controls, across CF survivors to CF non-survivors. Interestingly, LA dilatation was a bit more pronounced than RA dilatation, and in CF non-survivors, the LA volume index (33.7 ± 10.5 mL/m^2^) was almost equal to the cut-off value for LA dilatation in the general population (≥ 34 mL/m^2^). This shows that left heart must not be forgotten in the assessment of CF patients, and this evaluation cannot be based solely on the calculation of the LV ejection fraction. LA dilatation in our CF patients was associated with a significant increase in the mitral E/e’ ratio and a decrease in the mitral E/A ratio. Both parameters are associated with an increased LV filling pressure that could be explained by an increased LV preload, a low pulmonary blood flow, an increased pulmonary pressure, an impaired RV performance, and interventricular interdependence throughout interventricular septum [22].

Atrial phasic function is very important because it determines LV diastolic (dys)function [23]. There are three parts of the diastole which could be assessed with three different types of atrial function. LA reservoir function represents the ability of the LA to store pulmonary venous return during LV contraction and isovolumetric relaxation. Conduit LA function reflects the capability of transferring blood passively into the LV, whereas LA pump function means active contraction during the last phase of diastole and contributes to 15–30% of LV stroke volume. Similar refers to the RA and its phasic function. The only difference is that venous return comes from systemic and not pulmonary circulation.

LA conduit and reservoir functions assessed by strain were significantly lower in CF patients than in the control group. In fact, LA conduit function was impaired in CF patients, and LA booster pump function was similar between the observed groups. As the result, LA reservoir function was also impaired in CF patients comparing with controls. There was a trend of the gradual deterioration of the LA reservoir and conduit functions from controls across CF survivors to CF non-survivors. However, a statistical significance in LA function between CF survivors and non-survivors was not reached due to the limited sample size. The deterioration of LA conduit and reservoir functions in our study indicated the presence of LV diastolic dysfunction, even though parameters for LV diastolic dysfunction were still in normal range. However, it must be underlined that these differences in LA phasic function between CF patients and controls were obtained only in the four-chamber view and not in the two-chamber view, and, subsequently, they were also not observed in global values—obtained as the average between the four- and two-chamber views. These regional differences are not an exception because other authors also did not find a statistically significant difference in global LV and RV strains, but significant differences were found in some LV and RV segments [11,14]. This could be the result of segmental myocardial impairment in CF, but a more reasonable explanation is that this is the consequence of our small sample size.

RA phasic function was not significantly deteriorated in CF patients compared with controls. However, RA was significantly larger in CF patients than in controls. Sciatti et al. recently reported significantly larger RA area in CF patients than in controls [12]. RA volume was not analyzed in this study [12]. RA volume, similar to LA, gradually increased from controls to CF non-survivors. Our CF patients had significantly increased pulmonary pressure than controls, which could be one of the reasons for RA dilatation in this group.

The LA and RA volume indexes were significant predictors of mortality among our patients with CF. This showed the great importance of the evaluation of LA and RA volumes in the CF population. Besides the LA and RA volume indexes, the only predictors of mortality in the CF population were lung function parameters (FVC and FEV1). LA and RA strain parameters were not predictors of a lethal outcome in CF patients. The only independent predictor of mortality was lung function (FVC).

The most relevant clinical implication of the current study is underlined importance of a bi-atrial echocardiographic evaluation, which involves the assessment of the LA and RA volume indexes. This is not time-consuming and is widely available because an atrial volume assessment does not require any additional equipment besides a basic echocardiographic machine, which makes it favorable from a cost-effectiveness perspective. An additional evaluation of LA and RA strains would be beneficial and also recommended, because it provides better insight in atrial function and remodeling.

## 5. Limitations

Our study has several limitations. First, our sample size was limited and a statistical significance was not reached in many comparisons where obvious trend existed. Second, this was a retrospective study, and the echocardiographic exam was not optimized for the research, which resulted in the exclusion of some patients due to a lack of adequate images. Third, diabetes was very prevalent in CF patients and could contribute to atrial remodeling. However, CF induces diabetes in more than 50% of adult patients, and it also represents a characteristic of CF in a certain age group. Fourth, biomarkers related to RV or LV dysfunction were not available for this study, and these would have been helpful to reach a conclusion.

## 6. Conclusions

LA and RA were enlarged in CF patients. The dilatation was more remarkable in CF non-survivors than in CF survivors and controls. LA conduit and reservoir functions, evaluated by strain, were deteriorated in CF patients in comparison with controls. RA phasic function in CF patients was not significantly different from controls, even though there was an obvious trend of RA function impairment from controls to CF non-survivors. The LA and RA volume indexes, but not strain, were predictors of mortality in CF patients. This study underlines the importance of a bi-atrial assessment in CF patients, which involves the evaluation of, at least, LA global strain, as well as atrial volume indexes whenever is feasible. Further follow-up studies with larger numbers of patients are necessary to determine the predictive value of LA and RA phasic function and strain on the outcome in CF patients.

## Figures and Tables

**Table 1:** Demographic characteristics and clinical parameters of the study population.

	Controls (*n* = 32)	CF Survivors (*n* = 64)	CF Non-Survivors (*n* = 18)	*p*
Age (years)	36 ± 7	34 ± 11	34 ± 8	0.593
Female (%)	12 (38)	32 (50)	12 (67)	0.138
BMI (kg/m^2^)	24.0 ± 3.3	19.9 ± 3.7 ^b^	18.1 ± 2.1 ^a,c^	<0.001
Plasma glucose (mg/dL)	87 ± 13	133 ± 28 ^b^	146 ± 35 ^d^	0.011
Diabetes (%)	0 (0)	27 (42)	9 (50)	0.372
Urea (mg/dL)	26 ± 6	30 ± 7	44 ± 8 ^a,c^	0.007
Serum creatinine (mg/dL)	0.87 ± 0.18	0.87 ± 0.28	1.33 ± 0.54 ^c,d^	0.066
FVC (%)	-	63 ± 21	43 ± 17	<0.001
FEV1 (%)	-	45 ± 21	33 ± 13	0.033
MEF 25 (%)	-	21 ± 11	14 ± 7	0.310

BMI—body mass index, CF—cystic fibrosis, FVC—forced vital capacity, FEV1—forced expiratory volume in the first second, MEF 25—mean expiratory flow at 25% of the vital capacity. a—*p* < 0.01 for controls vs. CF non-survivors, b—*p* < 0.01 for controls vs. CF survivors, c—*p* < 0.05 for CF survivors vs. CF non-survivors, d—*p* < 0.05 for controls vs. CF non-survivors.

**Table 2:** Echocardiographic parameters of the study population.

	Controls (*n* = 32)	Cystic Fibrosis Survivors (*n* = 64)	Cystic Fibrosis Non-Survivors (*n* = 18)	*p*
LV parameters				
LV end-diastolic diameter (mm)	46.6 ± 0.5	41.2 ± 0.5 ^b^	42.8 ± 0.6 ^d^	<0.001
Interventricular septum thickness (mm)	9.0 ± 1.2	9.2 ± 1.6	9.3 ± 2.0	0.819
Posterior wall thickness (mm)	8.3 ± 1.9	8.9 ± 1.5	9.0 ± 2.0	0.300
Relative wall thickness	0.36 ± 0.08	0.43 ± 0.09 ^b^	0.43 ± 0.1 ^a^	<0.001
LV mass index (g/m^2^)	71.7 ± 19.7	73.8 ± 21.5	84.1 ± 35.1	0.202
Ejection fraction (%)	64 ± 6	63 ± 8	60 ± 11	0.257
E/A ratio	1.7 ± 0.7	1.3 ± 0.4 ^b^	1.2 ± 0.3 ^a^	<0.001
Deceleration time (ms)	215 ± 74	198 ± 68	211 ± 65	0.543
E/e’	5.9 ± 1.4	7.0 ± 1.8	8.8 ± 3.1 ^a,c^	<0.001
LV diastolic dysfunction (%)	3 (9)	7 (11)	8 (44) ^a,e^	0.001
LA volume index (mL/m^2^)	26.5 ± 5.3	26.6 ± 8.1	33.7 ± 10.5 ^a,e^	0.004
LA dilatation (%)	1 (3)	9 (14)	10 (56) ^a,e^	<0.001
RV parameters				
RV basal diameter (mm)	32.0 ± 4.6	29.9 ± 4.9 ^d^	29.6 ± 4.6	0.088
RV end-diastolic area (cm^2^)	16 ± 4	14 ± 4 ^d^	13 ± 3.5 ^f^	0.048
RV end-systolic area (cm^2^)	8.3 ± 2.5	7.6 ± 3.7	7.7 ± 3.6	0.686
Fractional area change (%)	48 ± 8	45 ± 10	47 ± 8	0.598
s’ (cm/s)	12.6 ± 1.4	11.6 ± 2.7	11.0 ± 1.5	0.191
TAPSE (mm)	24 ± 4	20 ± 4 ^b^	19 ± 3 ^a^	<0.001
RA volume index (mL/m^2^)	21.2 ± 5.5	23.1 ± 9.0 ^d^	24.9 ± 11.8 ^e^	0.010
RA dilatation (%)	5 (16)	11 (17)	7 (39)	0.096
PAPs (mmHg)	20 ± 7	31 ± 12 ^b^	35 ± 10 ^a^	<0.001
Pulmonary hypertension (%)	1 (3)	5 (8)	7 (39) ^a,e^	<0.001

A—late diastolic mitral flow (pulse Doppler), e’—the average early diastolic flow velocity across the septal and lateral segments of mitral annulus obtained by tissue Doppler, E—early diastolic mitral flow (pulse Doppler), LA—left atrial, LVEDD—left ventricle end-diastolic dimension, PAPs—systolic pulmonary pressure, RWT—relative wall thickness, LVMI—left ventricle mass index, RV—right ventricle, s’-systolic flow velocity across the lateral segment of tricuspid annulus, TAPSE—tricuspid annular plane systolic excursion. a—*p* < 0.01 for controls vs. CF non-survivors, b—*p* < 0.01 for controls vs. CF survivors, c—*p* < 0.05 for CF survivors vs. CF non-survivors, d—*p* < 0.05 for controls vs. CF survivors, e—*p* < 0.01 for CF survivors vs. CF non-survivors, f—*p* < 0.05 for. controls vs. CF non-survivors.

**Table 3:** Left atrial speckle tracking parameters.

	Controls (*n* = 32)	Cystic Fibrosis Survivors (*n* = 64)	Cystic Fibrosis Non-Survivors (*n* = 18)	*p*
**Global LA speckle tracking parameters**				
LA global strain (%)	38 ± 8	34 ± 9	33 ± 12	0.301
LA positive strain (%)	22 ± 8	19 ± 7	18 ± 8	0.228
LA negative strain (%)	15 ± 3	15 ± 5	15 ± 5	0.981
LA early diastolic strain rate (cm/s)	2.3 ± 0.7	1.9 ± 0.7 ^d^	1.7 ± 0.9 ^f^	0.047
LA late diastolic strain rate (cm/s)	2.3 ± 0.7	2.2 ± 0.9	2.0 ± 0.7	0.633
LA systolic strain rate (cm/s)	1.8 ± 0.4	1.9 ± 0.6	1.7 ± 0.5	0.638
**4Ch LA speckle tracking parameters**				
LA global strain (%)	38 ± 9	33 ± 10 ^d^	30 ± 12 ^f^	0.040
LA positive strain (%)	23 ± 9	19 ± 8 ^d^	15 ± 7 ^a^	0.014
LA negative strain (%)	15 ± 4	14 ± 6	15 ± 5	0.839
LA early diastolic strain rate (1/s)	2.5 ± 0.8	1.9 ± 0.8 ^b^	1.6 ± 0.8 ^a^	0.002
LA late diastolic strain rate (1/s)	2.1 ± 0.7	2.0 ± 0.9	1.9 ± 0.7	0.800
LA systolic strain rate (1/s)	1.7 ± 0.4	1.8 ± 0.8	1.6 ± 0.6	0.570
**2Ch LA speckle tracking parameters**				
LA global strain (%)	35 ± 7	34 ± 10	36 ± 12	0.544
LA positive strain (%)	20 ± 7	18 ± 8	21 ± 9.8	0.448
LA negative strain (%)	15 ± 4	16 ± 6	15 ± 5	0.998
LA early diastolic strain rate (1/s)	2.1 ± 0.7	1.8 ± 0.8	1.9 ± 0.9	0.398
LA late diastolic strain rate (1/s)	2.4 ± 0.9	2.4 ± 1.0	2.3 ± 0.8	0.876
LA systolic strain rate (1/s)	1.8 ± 0.5	1.9 ± 0.7	2.0 ± 0.6	0.831

2Ch—two chamber, 4Ch—four chamber, LA—left atrium. a—*p* < 0.01 for controls vs. CF non-survivors, b—*p* < 0.01 for controls vs. CF survivors, d—*p* < 0.05 for controls vs. CF survivors, f—*p* < 0.05 for controls vs. CF non-survivors.

**Table 4:** Right atrial speckle tracking parameters.

	Controls (*n* = 32)	Cystic Fibrosis Survivors (*n* = 64)	Cystic Fibrosis Non-Survivors (*n* = 18)	*p*
RA global strain (%)	38 ± 9	35 ± 13	35 ± 10	0.518
RA positive strain (%)	24 ± 8	21 ± 10	19 ± 7	0.251
RA negative strain (%)	14 ± 5	14 ± 6	16 ± 6	0.567
RA early diastolic strain rate (cm/s)	1.9 ± 0.6	1.7 ± 0.7	1.6 ± 0.7	0.528
RA late diastolic strain rate (cm/s)	2.1 ± 0.7	2.1 ± 0.9	2.6 ± 1.2	0.194
RA systolic strain rate (cm/s)	2.1 ± 0.6	2.1 ± 0.7	2.3 ± 0.9	0.672

CF—cystic fibrosis, RA—right atrium.

**Table 5:** Predictors of mortality in patients with cystic fibrosis.

Univariate			
	OR	95% CI	*p*
Age (years)	0.52	0.17–1.50	0.215
BMI (kg/m^2^)	0.83	0.67–1.00	0.056
FVC (%)	0.94	0.91–0.98	0.001
FEV1 (%)	0.96	0.93–0.99	0.042
LV mass index (g/m^2^)	1.01	0.99–1.03	0.153
LAVI (mL/m^2^)	1.09	1.02–1.16	0.008
LA longitudinal strain in 4Ch (%)	0.97	0.92–1.03	0.362
LA early diastolic strain rate 4Ch (cm/s)	0.59	0.28–1.24	0.161
LA positive strain 4Ch (%)	0.95	0.88–1.02	0.149
TAPSE (mm)	0.94	0.80–1.09	0.400
PAPs (mmHg)	1.02	0.98–1.07	0.335
RAVI (mL/m^2^)	1.10	1.02–1.18	0.020
RA longitudinal strain (%)	0.99	0.95–1.05	0.808

BMI—body mass index, FVC—forced vital capacity, LA—left atrium, LAVI—left atrial volume index, RA—right atrium, RAVI—right atrial volume index, TAPSE—tricuspid annular plane systolic excursion.
